# GATA3 functions downstream of BRCA1 to suppress EMT in breast cancer

**DOI:** 10.7150/thno.59280

**Published:** 2021-07-13

**Authors:** Feng Bai, Li-Han Zhang, Xiong Liu, Chuying Wang, Chenglong Zheng, Jianping Sun, Min Li, Wei-Guo Zhu, Xin-Hai Pei

**Affiliations:** 1 Guangdong Provincial Key Laboratory of Regional Immunity and Diseases, International Cancer Center, Marshall Laboratory of Biomedical Engineering, Shenzhen University Health Science Center, Shenzhen 518060, China.; 2 Department of Pathology, Shenzhen University Health Science Center, Shenzhen 518060, China.; 3 Dewitt Daughtry Family Department of Surgery, University of Miami, Miami, FL 33136, USA.; 4 School of Basic Medical Sciences, Lanzhou University, Lanzhou, Gansu, 730000, China.; 5 The Affiliated Cancer Hospital of Zhengzhou University, Zhengzhou, Henan 450008, China.; 6 The Second Affiliated Hospital of Xi'an Jiaotong University, Xi'an, Shaanxi, 710061, China.; 7 Department of Mathematics and Statistics, University of North Carolina at Greensboro, Greensboro, NC 27402, USA; 8 Department of Biochemistry and Molecular Biology, International Cancer Center, Shenzhen University Health Science Center, Shenzhen 518060, China.; 9 Department of Anatomy and Histology, Shenzhen University Health Science Center, Shenzhen 518060, China.

**Keywords:** BRCA1, GATA3, EMT, tumorigenesis, metastasis

## Abstract

**Purpose:** Functional loss of *BRCA1* is associated with poorly differentiated and metastatic breast cancers that are enriched with cancer stem cells (CSCs). CSCs can be generated from carcinoma cells through an epithelial-mesenchymal transition (EMT) program. We and others have previously demonstrated that BRCA1 suppresses EMT and regulates the expression of multiple EMT-related transcription factors. However, the downstream mediators of BRCA1 function in EMT suppression remain elusive.

**Methods:** Depletion of BRCA1 or GATA3 activates p18^I*NK4C*^, a cell cycle inhibitor which inhibits mammary epithelial cell proliferation. We have therefore created genetically engineered mice with Brca1 or Gata3 loss in addition to deletion of p18^I*NK4C*^, to rescue proliferative defects caused by deficiency of Brca1 or Gata3. By using these mutant mice along with human* BRCA1* deficient as well as proficient breast cancer tissues and cells, we investigated and compared the role of Brca1 and Gata3 loss in the activation of EMT in breast cancers.

**Results:** We discovered that BRCA1 and GATA3 expressions were positively correlated in human breast cancer. Depletion of BRCA1 stimulated methylation of GATA3 promoter thereby repressing GATA3 transcription. We developed Brca1 and Gata3 deficient mouse system. We found that Gata3 deficiency in mice induced poorly-differentiated mammary tumors with the activation of EMT and promoted tumor initiating and metastatic potential. Gata3 deficient mammary tumors phenocopied Brca1 deficient tumors in the induction of EMT under the same genetic background. Reconstitution of Gata3 in Brca1-deficient tumor cells activated mesenchymal-epithelial transition, suppressing tumor initiation and metastasis.

**Conclusions:** Our finding, for the first time, demonstrates that GATA3 functions downstream of BRCA1 to suppress EMT in controlling mammary tumorigenesis and metastasis.

## Introduction

Breast cancer is mainly divided into estrogen receptor (ER) positive luminal and ER-negative basal-like tumors 1. Basal-like breast cancers (BLBCs) are poorly differentiated and contain a number of distinct cell types which include cells that express luminal, basal, and mesenchymal biomarkers 2, 3. Consistently, we and others have demonstrated that at least some of the BLBCs originate from luminal epithelial cells 4-7. BLBCs are the most lethal, partly due to their enrichment of cancer stem cells (CSCs) that are thought to drive clinical relapse and metastasis 8. The CSCs can be generated from luminal tumor cells by an epithelial-mesenchymal transition (EMT) program, a process in which epithelial cells lose many of their epithelial characteristics and acquire mesenchymal features 4-9. The molecular mechanisms controlling EMT in breast cancers are poorly understood.

Mammary epithelia are mainly composed of luminal and basal cells that are maintained by luminal and basal progenitors, respectively 10. The maintenance of luminal cell fate is orchestrated by networks of transcription factors (TFs), including BRCA1 and GATA3 11. Interestingly, functional loss of *BRCA1* by germline or somatic mutation, or promoter methylation is associated with more than half of BLBCs 12-14. Loss of GATA3 expression is also associated with BLBCs and tumor metastasis 13, 15-19. Loss of function of BRCA1 in breast cancer cells enhances the expression of several EMT inducing transcription factors (EMT-TFs) including SLUG 5, TWIST 20, FOXC1/C2 21, and inversely reduces the expression of a few of the EMT suppressing TFs, such as FOXA1 22, 23, FOXO3 24, 25. However, no downstream mediator of BRCA1 in regulation of EMT in breast cancers has been identified. In cell line models, GATA3 functions as a tumor suppressor by inducing epithelial fates while suppressing mesenchymal fates 26-28. We previously demonstrated that BRCA1 suppresses EMT in breast cancer development 20. However, whether and how BRCA1 interacts with GATA3 to control EMT in mammary tumorigenesis remains elusive.

Due to growth and differentiation defects induced by Brca1 and Gata3 deficiency 15, 19, 29-32, mice deficient for either Brca1 or Gata3 rarely develop tumors, making it difficult to identify the role of Brca1 and Gata3 loss in regulating EMT in tumor development and metastasis. Targeted deletion of GATA3 in tumor cells leads to apoptosis preventing the analysis of the functional loss of GATA3 in tumor cell differentiation 33. Most, if not all, genetic studies have utilized co-deletion of one of the genes in the *p53* pathway to overcome the growth defects induced by *Brca1* deficiency in mice 5, 6, 29-31, 34. However, deletion of *p53* induces EMT and BLBCs 35-38, masking the contribution of *Brca1* depletion alone in these processes. Hence it is imperative that the role of Brca1 in controlling EMT and tumorigenesis be determined under a genetically intact *p53* background.

The RB protein which is phosphorylated and inactivated by CDK4 and CDK6 (CDK4/6), controls the G1-to-S transition of the cell cycle. CDK4/6 are inhibited by inhibitors of CDK4/6 (INK4) such as p16*^INK4A^ (p16)* and p18^INK4C^* (p18).* Inactivation of the INK4-CDK4/6-RB pathway (i.e. loss of INK4 or RB and amplification of cyclin D or CDK4/6) is a common event in breast cancers 13, 39. Most BRCA1-deficient breast cancers carry a dysfunctional INK4-CDK4/6-RB pathway 13, 40, 41, All widely used BRCA1 mutant breast cancer cell lines have deletions in either RB or p16 42, 43. We previously demonstrated that depletion of Brca1 or Gata3 activates p16/p18-CDK4/6-RB pathway leading to cell cycle arrest 7, 15, 44, 45 which was later confirmed by two independent groups 32, 46. Since inactivation of INK4-CDK4/6-RB pathway in mice induces mammary luminal type tumors with little effect on EMT activation 7, 15, 20, 47, p18;Brca1 and p18;Gata3 mutant mice provide us a unique opportunity to investigate and compare the role of Brca1 and Gata3 loss in activation of EMT in breast cancers under a genetically intact *p53* background.

In this report, we used p18, Brca1, and Gata3 singly and doubly deficient mouse models as well as human BRCA1 proficient and deficient cancer cells to determine the mechanisms underlying the role of BRCA1 in the regulation of GATA3 in mammary tumorigenesis and metastasis. We demonstrated that the depletion of BRCA1 stimulated methylation of GATA3 promoter thereby repressing GATA3 transcription. We found that Gata3 deficient mammary tumors phenocopied Brca1 deficient tumors in the induction of EMT and promotion of tumorigenesis and progression. We discovered that GATA3 functions downstream of BRCA1 to suppress EMT in controlling mammary tumor initiation and metastasis.

## Methods

### Mice, histopathology, and immunostaining

The generation of p18^-/-^, p18^+/-^, p18^-/-^;Brca1^MGKO^ (p18^-/-^;Brca1^f/f^;MMTV-Cre or p18^-/-^;Brca1^f/-^;MMTV-Cre), p18^-/-^;Brca1^+/-^, p18^+/-^;Brca1^+/-^, Gata3^f/f^, Gata3^+/-^, p18^+/-^;Gata3^+/-^, and p18^-/-^;Gata3^+/-^ mice has been previously described 7, 15, 20, 48. The Institutional Animal Care and Use Committee at the University of Miami and Shenzhen University approved all animal procedures. Histopathology and immunohistochemistry (IHC) were performed as previously described 7, 15, 20. The primary antibodies used were: BRCA1, E-cadherin (E-Cad), Vimentin (Vim), FRA1 (Cell Signaling), Fibronectin (Fn), TWIST, DNA Methyltransferase 1 (DNMT1) (Abcam), CD29, GATA3 (Santa Cruz). Immunocomplexes were detected using the Vectastain ABC DAB kit according to the manufacturer's instructions (Vector Laboratories). The positive results of IHC were quantified by H-score, as previously described 49, 50.

### Mammary tumor cell preparation, transplantation, and analysis of metastasis

Mammary tumors were dissected from female mice and tumor cell suspensions were prepared as previously described 7, 15, 20. For the transplantation of primary *p18^-/-^* and* p18^-/-^;Gata3^+/-^* mammary tumor cells, cells derived from mutant mice were inoculated into the left inguinal mammary fat pads (MFPs) of 6-week-old female NSG mice (Jackson Laboratory) with subcutaneous implantation of estrogen pellets. Four weeks after transplantation, animals were euthanized for analysis of histopathology and immunohistochemistry. For transplantation of *p18^-/-^;Brca1^MGKO^* tumor cells, cells that were infected with pBabe-puro-empty or pBabe-puro-Gata3 and selected in puromycin were then inoculated into the left and/or right inguinal MFPs of 6-week-old female NSG mice, respectively. Four weeks after transplantation, animals were euthanized and mammary tumors were dissected for histopathological, immunohistochemical, and biochemical analyses. For analysis of lung metastasis from mammary tumors, *p18^-/-^; Brca1^MGKO^* tumor cells that were infected with pBabe-puro-empty or pBabe-puro-Gata3 and selected in puromycin were inoculated into the MFPs of NSG mice. When newly generated tumors either reached the IACUC designated endpoint size (1.3 cm^3^; in 4-10 weeks) or the mice became moribund, the lungs were examined for detection of metastasis. For quantification of the number of metastatic nodules in the lungs, fixed lung tissues of all five lobes were sagittally sectioned at 200-μm intervals. At least three sections for each lobe were prepared and stained with H.E. The metastatic nodules in each lobe of lung tissue were confirmed by H.E. staining, counted under a microscope, and averaged. The number of nodules in all lobes was then calculated.

### Cell culture, treatment, transfection, and viral infection

MCF-7, T47D, MDA-MB-231, BT20, SUM149, and HCC1937 cells were cultured per ATCC recommendations. Primary murine mammary tumor cells were cultured in 10% FBS (Gibco). For drug treatment, cells were cultured in the presence of 5-aza-2'-deoxycytidine (DAC) or DMSO for 72 hours, and then were lysed for further analysis. For ectopic expression of BRCA1, HCC1937 and SUM149 cells were transfected with pBabe-empty or pBabe-HA-BRCA1, and for knockdown (KD) of BRCA1, MCF7 cells were infected with pGIPZ-empty or pGIPZ-shBRCA1, as previously described 20. For ectopic expression of Gata3, murine mammary tumor cells were infected with retroviruses expressing wild-type (WT) GATA3, pBabe-GATA3, or control pBabe vector, pBabe-empty, as previously described 15.

### Western blot, qRT-PCR, and flow cytometry

Western blot analysis was carried out as previously described 7, 15, 20, 51. Primary antibodies used are as follows: BRCA1, HSP90, GAPDH (Ambion), E-Cad, Vim, GATA3 (D13C9), (Cell Signaling), TWIST (Abcam). For qRT-PCR, total RNA was extracted using the RNeasy kit (Qiagen) according to the manufacturer's protocol and cDNA was generated using the Omniscript RT Kit (Qiagen). qRT-PCR was performed as previously reported 7. For surface marker analysis, tumor cells were isolated and stained. After exclusion of lymphocytes, dead cells, and Lin+ cells, the expression profile for CD24 and CD29 was determined by flow cytometry as previously described 15, 20.

### Methylation analysis

For methylation specific PCR (MS-PCR) analysis, genomic DNA extracted from cells were treated with bisulfite and analyzed for *GATA3* promoter methylation with specific primers amplifying the unmethylated or methylated allele as described 44, 52. Primer sequences were as below: FW: 5'-ACGATTTTCGATTTTTCGACGGTAGGAGTTTTTC-3' and RV: 5'-GACTATACTCGCGCCCTCTCGCCGA-3' for methylated *GATA3*. FW: 5'-ATGATTTTTGATTTTTTGATGGTAGGAGTTT-3' and RV: 5'-TCAACTATACTCACACCCTCTCA-3' for unmethylated* GATA3*.

### Human tumor samples and meta-analysis of data sets

Formalin fixed paraffin-embedded (FFPE) human breast cancer samples lacking patient-identifying information were obtained from the Tissue Bank Core Facility at the University of Miami as previously reported 20. Regions from tumor samples were microdissected and only samples with a consistent tumor cell content >75% of tissues were used for RNA extraction. The expression of genes was determined by qRT-PCR as previously reported 20. In addition, FFPE samples derived from BRCA1 WT and BRCA1 mutant patient-derived xenograft (PDX) tumors were prepared as previously reported 50.

For analysis of the correlation of *GATA3* mRNA with *BRCA1* mutation, the TCGA dataset with 974 cancer samples including 14 samples harboring *BRCA1* mutations was analyzed 13. For analysis of the correlation of *GATA3* methylation with* BRCA1* mutation, the TCGA dataset with 664 cancer samples including 13 samples harboring *BRCA1* mutations was analyzed.

### Statistical analysis

All data are presented as the mean ± SD for at least three repeated individual experiments for each group. Quantitative results were analyzed by two-tailed Student's t-test. P < 0.05 was considered statistically significant.

## Results

### Loss of BRCA1 abrogates GATA3 expression and activates EMT

**Figure 1:**
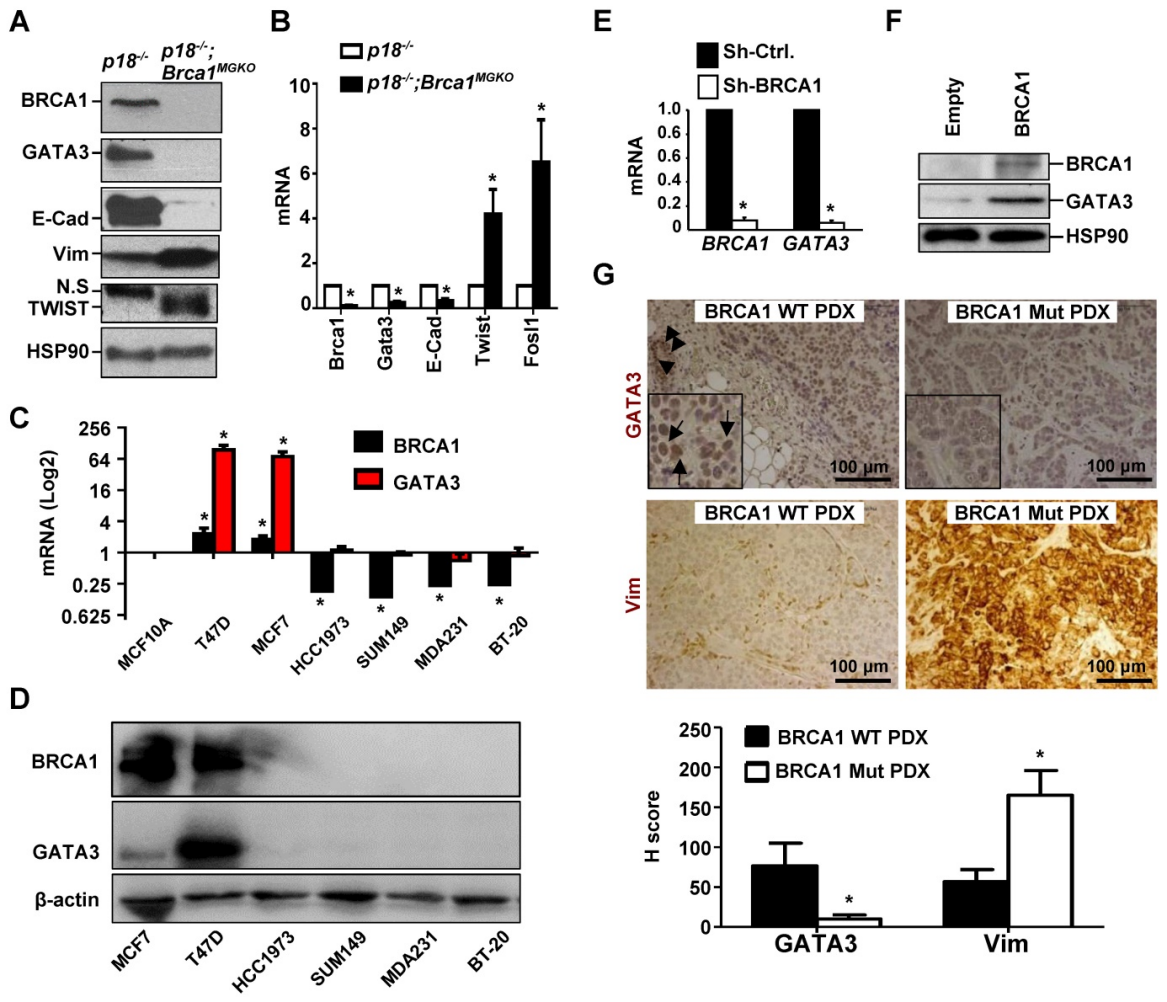
** BRCA1 positively regulates *GATA3* expression in breast cancer cells and tissues.** (A, B) Mammary tumor tissues (A) or primary tumor cells (B) from *p18^-/-^* and *p18^-/-^;Brca1^MGKO^* mice were analyzed by western blot (A) or qRT-PCR (B). N.S., non-specific band. Data in (B) represent the mean ± SD. from triplicates of two independent primary cell lines of each genotype. The asterisk (*) in (B) denotes a statistical significance from *p18^-/-^* and* p18^-/-^;Brca1^MGKO^* samples determined by the T-test. (C, D) The mRNA and protein levels of BRCA1 and GATA3 in human breast cell lines were detected by qRT-PCR (C) and Western blot (D). *BRCA1* and* GATA3* mRNA levels in breast cancer cell lines were normalized to that of MCF-10A cell line. Data in (C) represent the mean ± SD from triplicates of each of the two independent experiments. The asterisk (*) denotes a statistical significance from MCF-10 and breast cancer cell lines determined by the T-test. (E) T47D cells were infected with either pGIPZ-empty (sh-Ctrl) or pGIPZ-sh-BRCA1 (sh-BRCA1). Cells stably expressing sh-Ctrl or sh-BRCA1 were analyzed by qRT-PCR. Data represent the mean ± SD from triplicate of each of the two independent experiments. The asterisk (*) denotes a statistical significance from sh-Ctrl and sh-BRCA1 samples determined by the T-test. (F) HCC1937 cells were transfected with pBabe-empty (Empty) or pBabe-HA-BRCA1 (BRCA1). Expression of genes indicated were determined by western blot 48 hours after transfection. (G) PDX tumors generated by BRCA1 WT and BRCA1 mutant (Mut) breast cancers were stained with antibodies against GATA3 and Vim. GATA3 positive tumor cells (arrows in inset) and luminal epithelial cells (arrowheads) in mouse endogenous mammary glands are indicated. The H-scores for GATA3 and Vim in IHC were calculated. The results represent the mean ± SD of four individual tumors per group. The asterisk (*) denotes a statistical significance from BRCA1 WT PDX and BRCA1 Mut PDX samples determined by the T-test.

We previously found that heterozygous germline deletion of *Brca1* (*Brca1^+/-^*) or mammary epithelia specific deletion of *Brca1* (*Brca1^MGKO^*) in* p18*-deficient mice eliminated the expression of Gata3 in tumor-free mammary tissue and mammary epithelial cells (MECs) 7, 20. We then followed mammary tumors and found that *p18*^-/-^ tumors were well-differentiated tumors most of which were positive for GATA3 and E-Cad, a downstream target of GATA3, but *p18*^-/-^ tumors were negative or weakly expressing Vim and EMT-TFs which include TWIST and FRA1. Whereas, *p18^-/-^;Brca1^+/-^* and *p18^-/-^;Brca1^MGKO^* tumors were poorly differentiated tumors most of which were negative for GATA3 and E-Cad, but strongly positive for Vim and EMT-TFs (Figure 1A, Table 1, and in reference 15, 20). We isolated cells from primary mammary tumors and generated tumor cell lines, and consistently found that the expression of *Gata3* and* E-Cad* was abrogated and the expression of *Twist* and* Fosl1* (encoding Fra1) was enhanced in *p18^-/-^;Brca1^MGKO^* (Brca1 deficient) cells relative to the expression in *p18^-/-^* (Brca1 proficient) counterparts (Figure 1B, Figure S1A).

To consolidate the regulation of Gata3 by Brca1 in the control of EMT, we examined the expression of these genes in mouse embryos in which EMT plays a critical role during developmental stage 53. We found that the expression of *Gata3* along with *E-Cad* was significantly decreased in *p18*^-/-^;* Brca1*^MGKO^ embryos at both E9.5 and E12.5 of age relative to their *p18*^-/-^ counterparts (Figure S1B-C). We then knocked down *Brca1* in murine mammary epithelial cell line, HC11, and found that depletion of *Brca1* reduced the expression of *Gata3* and *E-cad*, but enhanced that of *Vim* (Figure S1D). Together, these data indicate deletion of *Brca1* in mice abrogates *Gata3* expression and activates EMT.

We then determined the expression of *GATA3* in human breast cancer cell lines and found that *GATA3* mRNA and protein levels were high in *BRCA1* proficient cells (T47D and MCF7) and low or undetectable in *BRCA1* deficient (BT20, MDA-MB231, SUM149, and HCC1937) cells (Figure 1C-D). Knockdown of *BRCA1* in T47D and MCF7 cells significantly reduced *GATA3* expression (Figure 1E and Figure 3F-G). Consistently, reconstitution of WT *BRCA1* in *BRCA1* mutant HCC1937 and SUM149 cells drastically induced GATA3 expression (Figure 1F, Figure S2). We then performed IHC analysis and found that GATA3 expression was barely detectable in *BRCA1* mutant PDX tumors, but readily detected in *BRCA1* WT PDX tumors, whereas Vim was highly expressed in *BRCA1* mutant PDX tumors (Figure 1G). When the number and intensity of positive cells were quantified, we found that the H scores for GATA3 in BRCA1 mutant tumor cells were significantly less than the H scores in BRCA1 WT counterparts, whereas, the H scores for Vim in BRCA1 mutant tumor cells were significantly more than the H scores in BRCA1 WT counterparts (Figure 1G, bottom panel). These results confirm that BRCA1 is required to maintain GATA3 expression in human breast cancer cells.

### Expression of BRCA1 is positively correlated with that of GATA3 in human breast cancers

**Figure 2:**
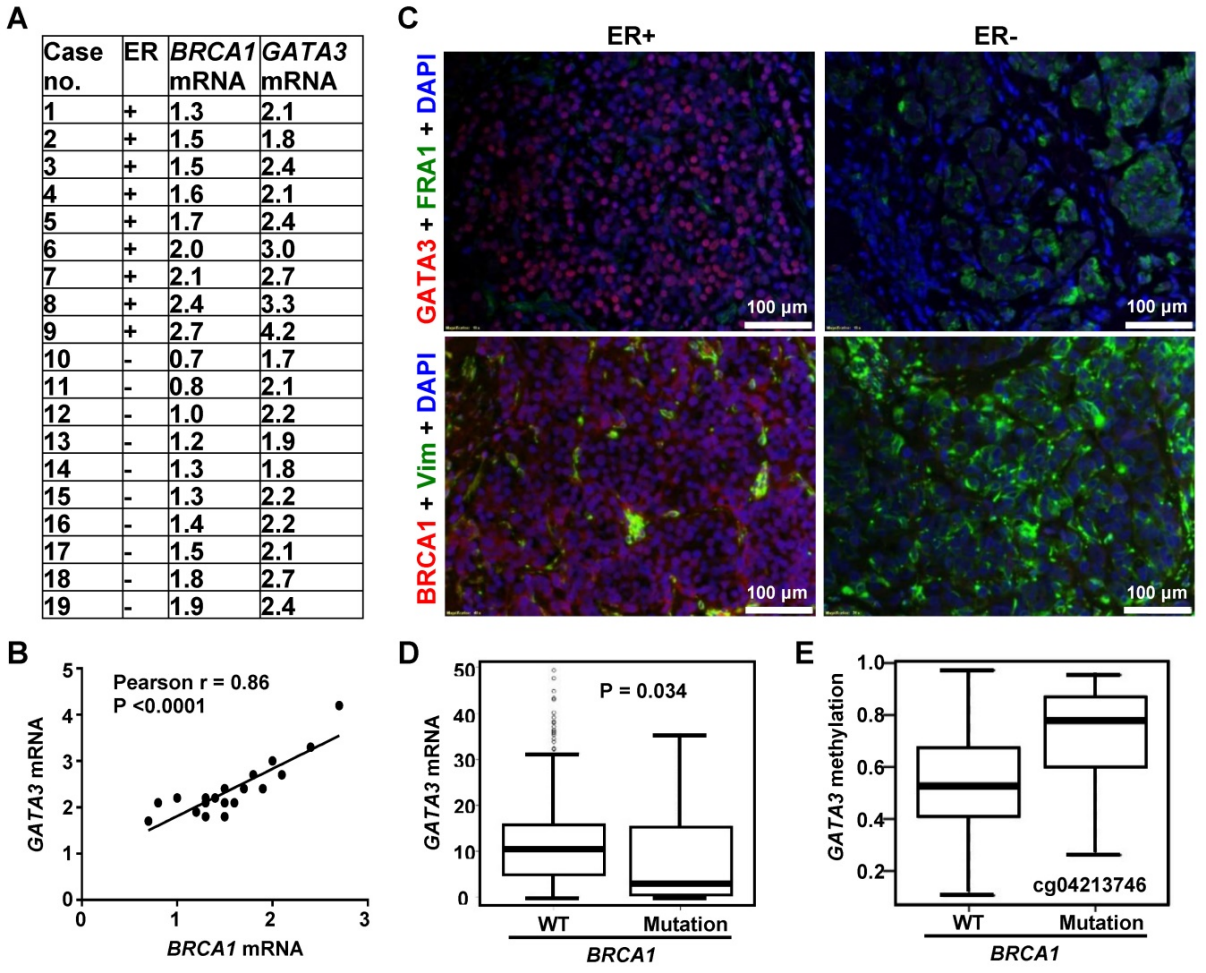
** Correlation analysis of BRCA1 with GATA3 in human breast cancers.** (A) Summary of expression of *BRCA1* and* GATA3* by qRT-PCR. The levels of *BRCA1* and* GATA3* mRNA are expressed relative to the corresponding values for T47D luminal tumor cell, as we previously reported 20. (B) Correlation analysis of* BRCA1* and* GATA3* mRNA levels for breast cancer sample. (C) Representative immunostaining analysis for human breast cancer samples. Case# 8 (ER+) and case#10 (ER-) in (A) were selected for analysis. (D, E) Correlation analysis of *GATA3* mRNA (D) and *GATA3* promoter methylation (E) levels between breast cancers with *BRCA1* WT and mutations in the TCGA dataset. Note, methylation levels in all 7 CpGs tested in *GATA3* promoter are higher in *BRCA1* mutants than in WTs. Representative methylation level in a CpG is shown.

Prompted by the findings derived from mouse models, we then employed human breast cancer samples to determine the correlation of BRCA1 and GATA3. By using our previously published resource of 43 invasive human breast cancers and the RNA prepared from microdissected FFPE sections of tumors 20, we assessed* BRCA1* and* GATA3* mRNA levels in 9 ER-positive and 10 ER-negative samples. *BRCA1* and *GATA3* mRNA levels were significantly higher in ER-positive tumors when compared to ER-negative tumors (2.67 ± 0.74 vs. 2.13 ± 0.29, p = 0.049 for *GATA3*; 1.87 ± 0.47 vs. 1.29 ± 0.39, p = 0.009 for* BRCA1*). Importantly, across both ER-negative and ER-positive tumors, we observed a significant relationship between *GATA3* mRNA and *BRCA1* mRNA levels (Figure 2A-B). We then performed immunostaining for these human breast cancer samples and found that in the ER-positive group, BRCA1-positive tumors whose *BRCA1* mRNA levels were equal to or higher than 1.5 expressed moderate to strong GATA3 protein. Conversely the human breast samples in ER-negative group, BRCA1-negative tumors whose *BRCA1* mRNA levels were less than 1.5 expressed weak or undetectable GATA3 protein (Figure 2C, Figure S3, and data not shown). Together, these clinical findings are consistent with the results from our mice cohorts; thereby suggesting an opportunity to use murine systems to further explore how BRCA1 interacts with GATA3 to control EMT in breast cancers.

### BRCA1 mutation is associated with GATA3 promoter hypermethylation and reduced GATA3 expression in human breast cancers

We performed data mining in the TCGA breast cancer dataset. A one-sided Wilcoxon rank sum test was conducted to test the null hypothesis (*GATA3* mRNA level is greater in *BRCA1* mutant tumors than in WT tumors) versus the alternative hypothesis (*GATA3* mRNA level is less in *BRCA1* mutant tumors than in WT tumors). The resulted p-value (0.034 < 0.05) suggested that we should reject the null hypotheses and conclude that *GATA3* mRNA level is significantly less in* BRCA1* mutant tumors than in *BRCA1* WT tumors (Figure 2D). Notably, median methylation levels in all 7 CpGs tested with the *GATA3* promoter are higher in *BRCA1* mutants than in WTs (Figure 2E, Figure S4). For each CpG site, Wilcoxon rank sum test was conducted to test the difference of methylation levels between *BRCA1* mutants and WTs. The resulted p-values were then adjusted to correct for multiple testing by controlling overall false discover rate at 0.05. However, no CpGs were significant after adjustment, likely due to the limited number of *BRCA1* mutant samples (13 only available). These data indicate that *BRCA1* mutation is associated with *GATA3* promoter hypermethylation and reduced *GATA3* expression in human breast cancer samples.

### Depletion of BRCA1 enhances methylation of GATA3 promoter

**Figure 3:**
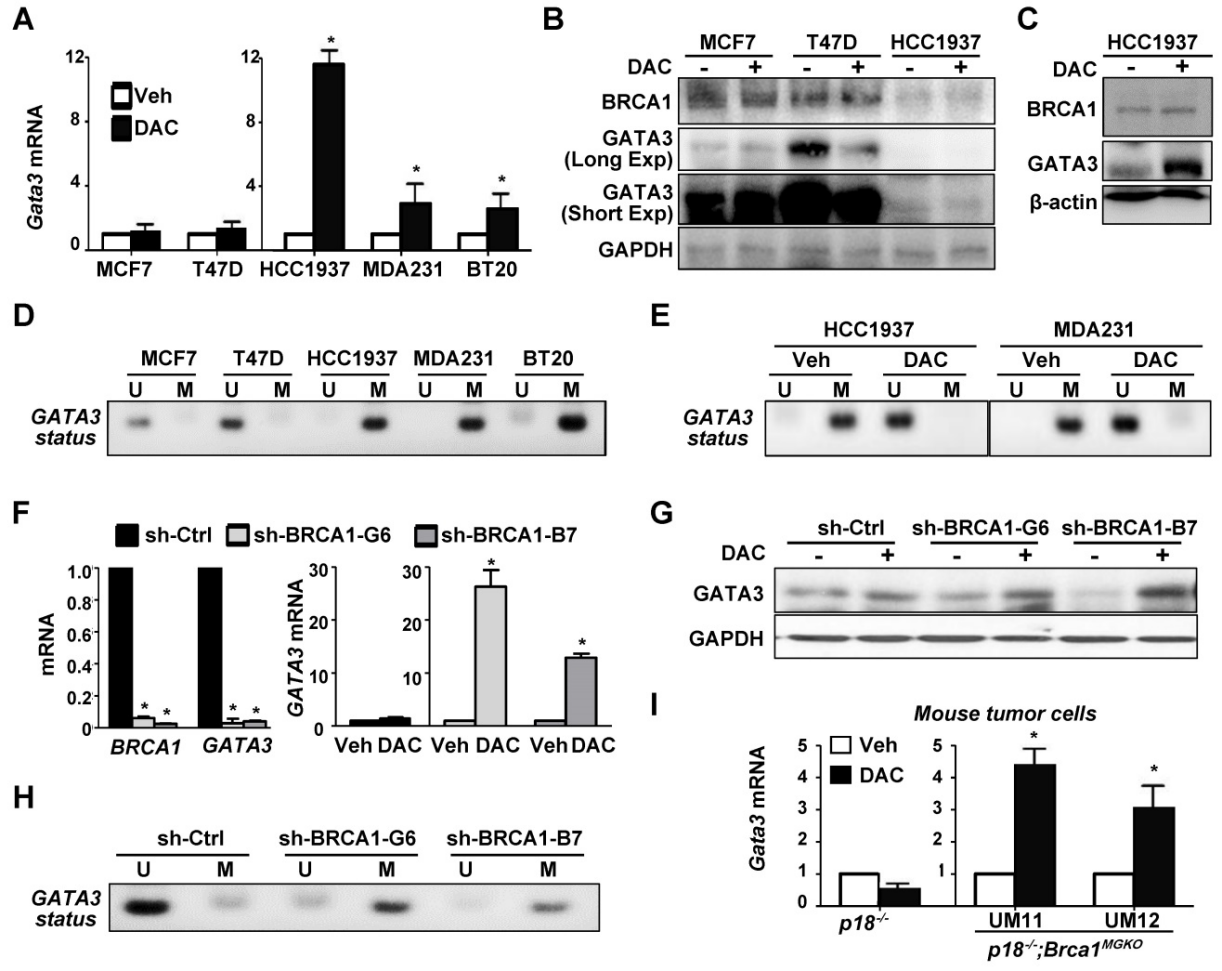
***GATA3* gene is hypermethylated in *BRCA1*-deficient cells, and depletion of *BRCA1* in *BRCA1* proficient cells stimulates methylation of *GATA3* gene.** (A, B, C) Human breast cancer cells were treated with either DMSO (Veh) or DAC (5 µM) for 72 hours, and then analyzed by qRT-PCR (A) and western blot (B, C). Triple the amount of protein lysates from HCC1937 in (B) were analyzed in (C). MDA231, MDA-MB-231. Note the drastic increase of *GATA3* mRNA and protein in DAC-treated HCC1937 cells. (D, E) MS-PCR analysis of *GATA3* promoter methylation for a panel of cell lines (D), as well as HCC1937 and MDA231 cells treated with Veh or DAC for 72 hours (E). U, unmethylated; M, methylated. (F, G, H) MCF7 cells infected with either pGIPZ-empty (sh-Ctrl) or pGIPZ-shBRCA1 targeting different sequences of human *BRCA1* (sh-BRCA1-G6, and sh-BRCA1-B7) were treated with or without DAC for 72 hours and then analyzed by qRT-PCR (F), western blot (G), or MS-PCR analysis of *GATA3* promoter methylation (H). (I) *p18^-/-^* and* p18^-/-^;Brca1^MGKO^* mammary tumor cells were treated with either Veh or DAC (5 µM) for 72 hours, and then analyzed by qRT-PCR. (UM11) and (UM12) represent two independent primary cell lines derived from two individual mice (*p18^-/-^;Brca1^MGKO^*). Data in (A), (F), and (I) represent the mean ± SD from triplicates of each of the two independent experiments. The asterisk (*) denotes a statistical significance from DAC and Veh treated samples, or from sh-Ctrl and sh-BRCA1-G6 or sh-BRCA1-B7 samples determined via student T-test.

Prompted by the finding that *BRCA1* mutation is associated with enhanced methylation of the* GATA3* promoter and reduced expression of *GATA3*, we hypothesized that loss of function of *BRCA1* stimulates methylation of *GATA3* promoter thereby suppressing its transcription. To test this hypothesis, we treated breast cancer cells with 5-aza-2'-deoxycytidine (DAC), a DNMT inhibitor, and found that *GATA3* mRNA levels in all *BRCA1*-deficient cells, HCC1937, MDA-MB-231, and BT20, were enhanced more than 2.5 fold, whereas *GATA3* mRNA levels in *BRCA1*-proficient cells, MCF7 and T47D, were enhanced less than 1.5 fold (Figure 3A). Consistently, western blot analysis revealed that DAC treatment did not increase GATA3 protein levels in MCF7 and T47D cells, whereas GATA3 protein levels in *BRCA1* mutant HCC1937 cells were clearly enhanced, though the basal levels of GATA3 were low (Figure 3B-C). Interestingly, BRCA1 levels showed insignificant change in response to DAC treatment. We performed MS-PCR and confirmed that *GATA3* promoter in HCC1937, MDA-MB-231, and BT20 cells was hypermethylated, whereas *GATA3* promoter in MCF7 and T47D cells was unmethylated (Figure 3D). We also treated HCC1937 and MDA-MB-231 cells with DMSO and DAC and found that DAC, not DMSO, converted hypermethylated *GATA3* promoter into its un-methylated form (Figure 3E). These results not only confirm the specificity of the MS-PCR for detection of hypermethylated *GATA3* promoter, but also demonstrate the activity of DAC in the de-methylation of *GATA3* promoter. Further, these data indicate that *BRCA1* deficiency is indeed associated with methylation of *GATA3* promoter. We knocked down *BRCA1* in MCF7 cells using *BRCA1* shRNA to target two different sequences. We found that treatment of *BRCA1*-KD, but not control-KD, MCF7 cells with DAC led to drastic increase of *GATA3* mRNA and protein levels relative to Veh treatment (Figure 3F-G). MS-PCR analysis revealed that the *GATA3* promoter in *BRCA1*-KD MCF7 cells was hypermethylated relative to the *GATA3* Promotor in control MCF7 cells (Figure 3H). These results indicate that depletion of *BRCA1* in breast cancer cell lines enhances methylation of the *GATA3* promoter thereby repressing its transcription.

To genetically confirm the role of *Brca1* in regulating the methylation of *Gata3* gene we treated *p18^-/-^* (Brca1 proficient) and *p18^-/-^;Brca1^MGKO^* (Brca1 deficient) tumor cells with DAC. We found that DAC drastically enhanced *Gata3* expression in *p18^-/-^;Brca1^MGKO^*, but not in *p18^-/-^*, tumor cells (Figure 3I). These results suggest that *Gata3* promoter is hypermethylated in* Brca1* deficient cells relative to that in *Brca1* proficient cells, which confirms our findings derived from human breast cancer samples and cells. Since loss of *p18* is required for *Brca1* deficient mammary tumorigenesis 7, 20 and inactivation of the INK4-CDK4/6-RB pathway stimulates transcription of DNMT1 in a few cell lines 54, we performed IHC and consistently detected increased DNMT1 expression in *p18*-null MECs and stromal cells relative to DNMT1 expression in their WT counterparts (Figure S5). These data suggest that *p18* loss stimulates DNMT1 expression in mammary gland, which promotes methylation of the *Gata3* promoter when *Brca1* is deficient. Together with the finding that mammary tumors developed in *p18* deficient mice are *Brca1*- and *Gata3*-positive well-differentiated tumors (Figure 1, and Reference 7, 15, 20), these results indicate that *Brca1* protects the *Gata3* promoter from methylation to maintain *Gata3* transcription in the suppression of mammary tumors.

### Heterozygous germline deletion of Gata3 in mice leads to poorly-differentiated mammary tumors with activation of EMT

**Figure 4:**
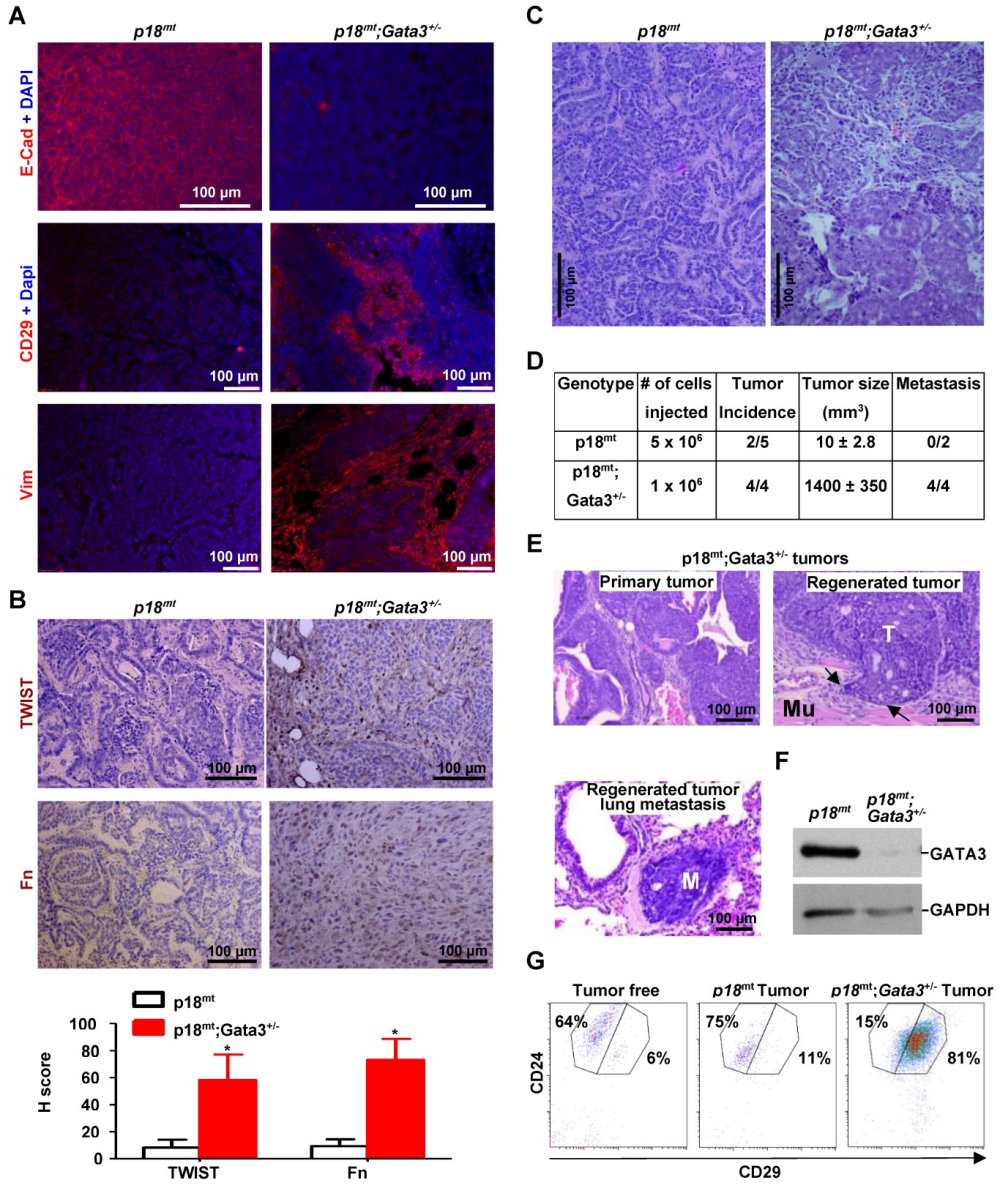
*** Gata3* deficiency in mice induces poorly differentiated mammary tumors with activation of EMT, and *Gata3* deficient tumor cells harbor the enhanced tumor initiating and metastatic potential.** (A, B, C) Representative IF staining (A), IHC (B), and H & E (C) analysis of primary mammary tumors developed in mice with the indicated genotypes. The H-scores for TWIST and Fn in (B) were calculated. The results represent the mean ± SD of four individual tumors per group. The asterisk (*) denotes a statistical significance from* p18^mt^* and* p18^mt^;Gata3^+/-^* samples determined by the T-test. (D) Primary tumor cells were transplanted into MFPs of NSG mice with estradiol supplement. Four weeks later, recipient mice were dissected, regenerated mammary tumors were counted, and metastasis in lungs was determined. Two mice that received *p18^mt^;Gata3^+/-^* tumor cell transplants died of lung metastasis in 4 weeks. (E) H & E. staining of *p18^mt^;Gata3^+/-^* primary and regenerated mammary tumors, as well as lung metastasis from regenerated mammary tumors. Note the regenerated mammary tumor (T) invasion into surrounding muscle (Mu) and metastasis (M) in the lung. (F, G) Representative tumors generated by transplantation of *p18^mt^;Gata3^+/-^* or* p18^mt^* tumor cells into mammary fat pad were analyzed by western blot (F) and FACS (G).

**Figure 5:**
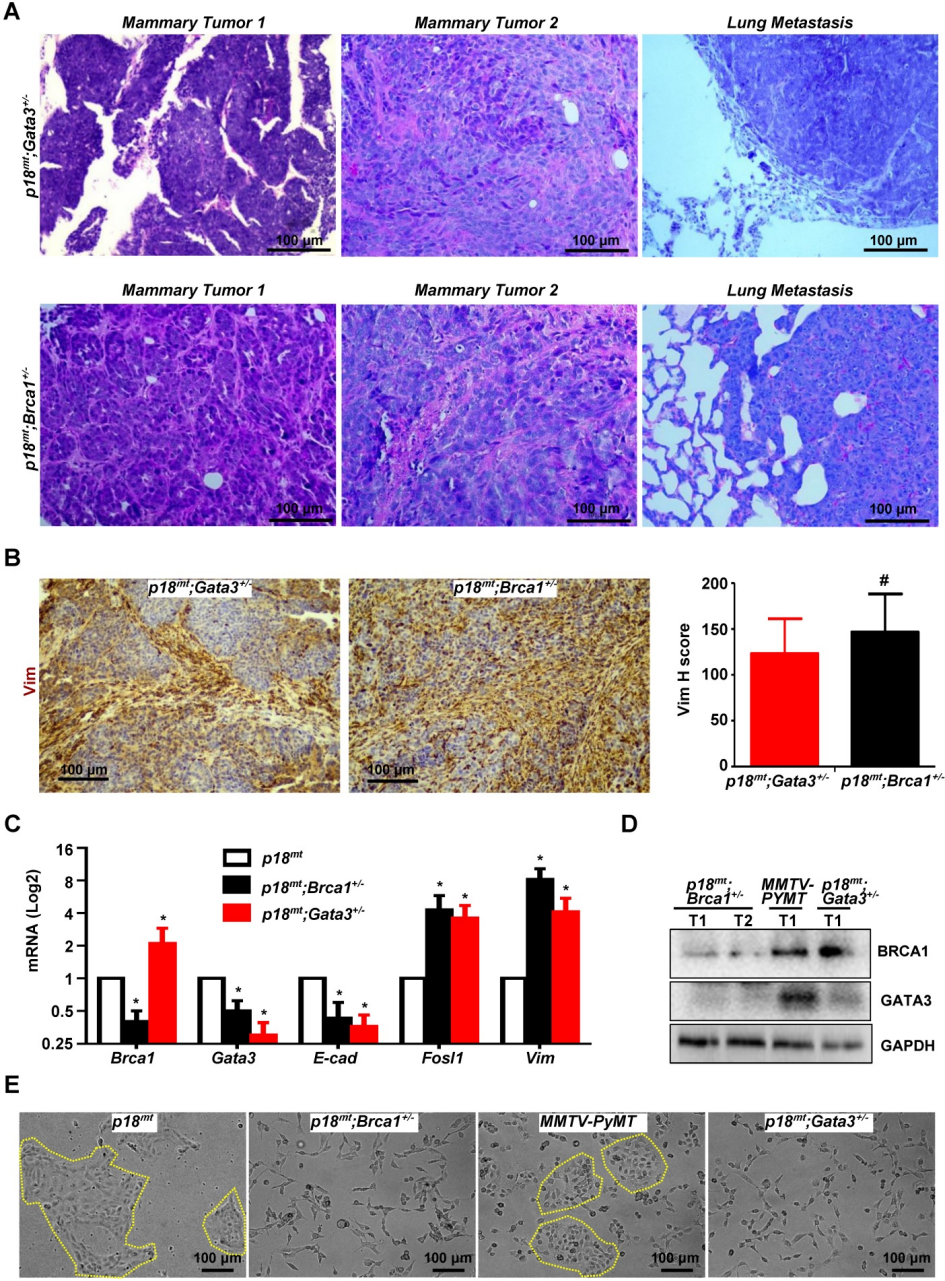
***Gata3* deficient mammary tumors phenocopy* Brca1* deficient tumors in induction of EMT.** (A, B) Representative mammary tumors and their lung metastasis were analyzed and compared by H.E (A) and IHC (B). Two independent mammary tumors (Mammary Tumor 1 and Mammary Tumor 2) and one lung metastasis derived from *p18^mt^;Gata3^+/-^* and* p18^mt^;Brca1^+/-^* mice individually are shown in (A). The H-scores for Vim in (B) were calculated. The results represent the mean ± SD of four individual tumors per group. The number sign (#) denotes a statistical insignificance from *p18^mt^;Gata3^+/-^* and* p18^mt^;Brca1^+/-^* samples determined by the T-test. (C, D, E) The expression of genes and the morphology of the tumor cell lines derived from primary mammary tumors of different genotype were determined by qRT-PCR (C), western blot (D), and phase contrast microscopy (E). Data in (C) represent the mean ± SD. from triplicates of two independent primary cell lines of each genotype. The asterisk (*) in (C) denotes a statistical significance from *p18^mt^* and* p18^mt^;Gata3^+/-^* or* p18^mt^;Brca1^+/-^* samples determined by the T-test.

Given the direct regulation of GATA3 by BRCA1 shown above, and EMT-suppressive function of ectopic GATA3 in cell lines 26-28, we hypothesized that depletion of *Gata3* in mice resulted in poorly differentiated mammary tumors with the induction of EMT which phenocopied *Brca1*-deficient tumors. To test this hypothesis, we generated *p18^-/-^, p18^+/-^, Gata3^+/-^, p18^-/-^;Gata3^+/-^,* and* p18^+/-^;Gata3^+/-^* mice in Balb/c-B6 mixed background. Due to the haploinsufficient role of p18 in mammary tumor development 7, 55 and the indistinguishable mammary tumor phenotype between* p18^-/-^* and* p18^+/-^*, as well as *p18^-/-^;Gata3^+/-^* and* p18^+/-^;Gata3^+/-^* mice, we combined *p18^-/-^* and* p18^+/-^* mice as *p18^mt^* group, *p18^-/-^;Gata3^+/-^* and* p18^+/-^;Gata3^+/-^* mice as *p18^mt^;Gata3^+/-^* group. We followed tumor development in these mice and found that 50% (n = 34) of *p18^mt^;Gata3^+/-^* and 30% (n = 27) of *p18^mt^* mice developed mammary tumors between 8-20 months, indicating that haploid loss of *Gata3* in p18 mutant mice accelerates mammary tumorigenesis (Table 2). Though the mammary tumor incidence of* p18^mt^* mice in the Balb/c-B6 mixed background was lower when compared to the Balb/c background, as we previously reported 15, *p18^mt^* mammary tumors in a Balb/c-B6 mixed background were also E-cad positive, well-differentiated mammary tumors (Table 2, Figure 1, Figure 4, and data not shown). *p18^mt^;Gata3^+/-^* mammary tumors displayed typical pathological characteristics of poorly-differentiated tumors--highly heterogeneous cell types with increased necrosis, squamous metaplasia, spindle cells, nuclear-cytoplasm ratio, and mitotic indices (Table 2, Figure 4C, E, Figure 5A). 77% (13 out of 17) of *p18^mt^;Gata3^+/-^* mammary tumors were positive for EMT markers including fibronectin (Fn), vimentin (Vim), CD29, and EMT-TFs in 2-60% of the tumor cells. Whereas only a quarter (2 out of 8) of* p18^mt^* tumor was positively stained with mesenchymal markers in ~2% of the tumor cells (Table 2, Figure 4A-B, Figure 5B, Figure S6). Although 29% (5 out of 17) of p*18^mt^;Gata3^+/-^* mammary tumors and none (0 out of 8) of *p18^mt^* mammary tumors metastasized to lung, no significant increase of metastasis was observed in *p18^mt^;Gata3^+/-^* tumors relative to *p18^mt^* tumors when statistical analysis was conducted. This was partially caused by the development of kidney cysts and consequential renal failure, as well as various types of tumors in other organs including lymphoma and sarcoma in *p18^mt^;Gata3^+/-^* mice (Reference 48, and data will be published elsewhere), which prevented a thorough analysis of mammary tumor related metastasis. Together, these results indicate that haploid loss of *Gata3* induces poorly-differentiated mammary tumors with the activation of EMT.

### Gata3 deficiency promotes tumor initiating and metastatic potential

We transplanted primary tumor cells into mammary fat pads (MFPs) of NSG mice and found that 2 out of 5 mice received 5 x 10^6^ *p18^mt^* tumor cell transplants produced tiny tumors (10 ± 2.8 mm^3^ in size) in 4 weeks. Regenerated* p18^mt^* mammary tumors, like primary *p18^mt^* tumors, were well differentiated tumors with typical epithelial features and markers, but with no metastasis (Figure 4A-D, Table 1, Table 2, and Ref 50). Surprisingly, in the same time period all mice (4 out of 4) that received 1 x 10^6^ *p18^mt^;Gata3^+/-^* tumor cell transplants developed huge mammary tumors (1440 ± 350 mm^3^ in size) and exhibited clinical sign of dyspnea (Figure 4D, E). Two mice that received *p18^mt^;Gata3^+/-^* tumor cell transplants died of lung metastasis in 4 weeks. Pathological analysis revealed that, like primary *p18^mt^;Gata3^+/-^* mammary tumors, regenerated *p18^mt^;Gata3^+/-^* mammary tumors were poorly differentiated, highly aggressive, invaded into the surrounding muscles, and metastasized to the lungs (Figure 4D-E). FACS analysis revealed that regenerated *p18^mt^;Gata3^+/-^* mammary tumors were composed of predominantly (81%) CD24^+^CD29^high^ cells, previously demonstrated as CSC-enriched cell populations 56, 57, while regenerated *p18^mt^* tumors were composed of predominantly (75%) CD24^+^CD29^low^ luminal/epithelial cells (Figure 4G), which was also detected in primary *p18^mt^* tumors 20. The regenerated *p18^mt^;Gata3^+/-^* tumors expressed almost no detectable Gata3 relative to regenerated *p18^mt^* tumors (Figure 4F), suggesting that the regenerated tumors were enriched for cells with complete loss of Gata3. Notably, both primary and regenerated Brca1 deficient mammary tumors were also composed of predominantly CD24^+^CD29^high^ cells, and *Brca1* deficient mammary tumor cells harbored drastically enhanced potential for tumor initiation and metastasis 20, 50. Together, these results indicate that *Gata3* deficiency in mammary tumor cells promotes the potential for tumor initiation and metastasis.

### Gata3 deficient mammary tumors phenocopy Brca1 deficient tumors in induction of EMT

Haploid loss of *Gata3* activates EMT and promotes mammary tumorigenesis and metastasis, suggesting that *Gata3* deficient tumors phenocopy *Brca1* deficient tumors. To test this hypothesis, we compared the mammary tumors developed in *Gata3^+/-^* and* Brca1^+/-^* mice under the same *p18^mt^* background. Pathological analysis revealed that *p18^mt^;Gata3^+/-^* mammary tumors were highly heterogeneous and with various degrees of whorls and clusters of spindle-shaped cells, which are typical morphological characteristics of mesenchymal cells and also frequently observed in *p18^mt^;Brca1^+/-^*mammary tumors (Figure 4C, 5A, and Ref 7). Importantly, when compared with *p18^mt^* tumors, both *p18^mt^;Gata3^+/-^*and* p18^mt^;Brca1^+/-^*mammary tumors more frequently metastasized to the lungs, and the lung metastasis were also enriched with mesenchymal-like cells. We examined EMT markers including Vim, Fn, E-cad, and EMT-TFs and found that all EMT markers positively stained in *p18^mt^;Brca1^+/-^* primary mammary tumors and its related metastasis were also detected in *p18^mt^;Gata3^+/-^*counterparts. Interestingly, very weak Brca1 and Gata3 expression were observed in less than 10% of *p18^mt^;Brca1^+/-^* lung metastatic tumor cells, while Gata3 was barely detectable in *p18^mt^;Brca1^+/-^* lung metastasis. (Table 1, Table 2, Figure 4A-B, Figure 5B, Fig. S6, S7, and Ref 50). When the number and intensity of positive cells were quantified, we found that the H scores for Vim in *p18^mt^;Gata3^+/-^*tumor cells were comparable with the H scores in *p18^mt^;Brca1^+/-^* counterparts (Figure 5B, right panel).

To determine the similarity of cellular phenotype of *Brca1* and* Gata3* deficient tumor cells, we generated five *p18^mt^;Brca1^+/-^,* five* p18^mt^;Gata3^+/-^,* and three *p18^mt^* tumor cell lines, each of which was derived from a primary mammary tumor developed in an individual mouse. In addition, due to the slow proliferation rate of *p18^mt^* tumor cells in vitro, we isolated and characterized three cell lines from mammary tumors developed in MMTV-PYMT mice, which have been well characterized as luminal type mammary tumor model with genetically intact *Brca1* and* Gata3* 33, 58. We confirmed the reduction of *Brca1* and* Gata3* expressions in *18^mt^;Brca1^+/-^* and *p18^mt^;Gata3^+/-^* cells, respectively, when compared with those in *p18^mt^* and MMTV-PYMT cells (Figure 5C-D). Importantly, we noticed that *Brca1* mRNA and protein levels were not decreased, rather slightly increased in *p18^mt^;Gata3^+/-^* cells relative to *p18^mt^* and MMTV-PYMT cells, whereas *Gata3* mRNA and protein levels were, again, significantly reduced in *p18^mt^;Brca1^+/-^* and* p18^mt^;Gata3^+/-^* cells relative to *p18^mt^* and MMTV-PYMT cells (Figure 5C-D). To directly test if Gata3 loss impacts the expression of *Brca1*, we generated *Gata3^f/f^* MECs and transduced them with pMX-Cre and pMX-Empty. We found that deletion of *Gata3* in MECs did not cause significant change of *Brca1* mRNA level (Figure S8A). We demonstrated that knockdown of* GATA3* in T47D human breast cancer cells or overexpression of WT *Gata3* in *Gata3* deficient mouse mammary tumor cells did not cause significant change of *BRCA1* mRNA levels (Figure S8B-C). These data further support that Gata3 is downstream of Brca1, and that Brca1 is less likely a target of Gata3 in mammary epithelial and cancer cells. Consistent with the findings derived from tumor tissues, primary *p18^mt^;Brca1^+/-^* and* p18^mt^;Gata3^+/-^* tumor cells also exhibited a typical mesenchymal-like morphology with a high level of EMT markers, whereas, epithelial-like morphology were observed in *p18^mt^* and MMTV-PYMT tumor cells (Figure 5C, E). Taking into consideration of the similar potential of p*18^mt^;Brca1^+/-^* and* p18^mt^;Gata3^+/-^* tumor cells in promoting tumor initiation and metastasis, these results indicate that in breast cancer cells *Gata3* deficiency functions similarly with *Brca1* deficiency in activation of EMT and promotion of tumorigenesis and metastasis.

### Reconstitution of Gata3 in Brca1-deficient tumor cells activates mesenchymal-epithelial transition (MET) suppressing tumor initiation and metastasis

**Figure 6:**
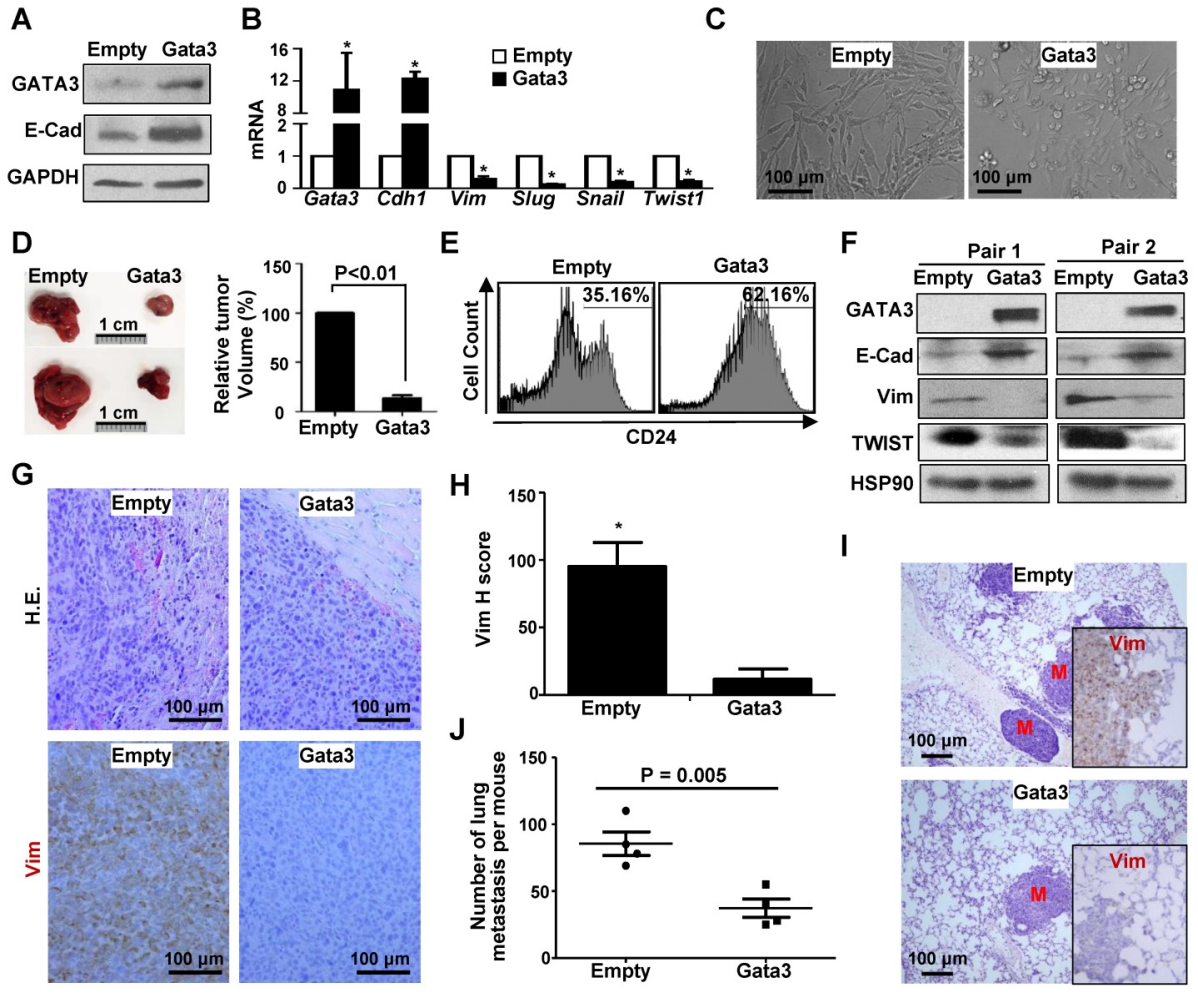
** Ectopic *Gata3* in *Brca1*-deficient tumor cells activates MET suppressing tumorigenesis and metastasis.** *(*A-C) *p18*^-/-^;*Brca1*^MGKO^ tumor cells were infected with pBabe-Empty (Empty) or pBabe-Gata3 (Gata3) and selected in puromycin. The expression of genes (A, B) and cell morphology (C) were determined. Data in (B) represent the mean ± SD. from triplicates of two independent experiments. The asterisk (*) denotes a statistical significance from Empty and Gata3 samples determined by the T-test. (D) 2×10^6^ Empty and Gata3-expressing *p18*^-/-^;*Brca1*^MGKO^ cells were transplanted into left and right inguinal MFP of NSG mice, respectively, in a pairwise manner. Four weeks later, regenerated tumor volumes were determined. The relative volumes of tumors generated by Gata3-expressing cells to tumors produced by Empty-expressing cells in each mouse were calculated and averaged. Data in the right represented as mean ± SD of five tumors in each group. Representative gross pictures of tumors were shown in the left. (E-G) Tumors generated from (D) were analyzed by flow cytometry (E), western blot (F), as well as H.E. and IHC staining (G). Note drastically decreased expression of Vim and increased that of E-cad in Gata3-expressing tumors relative to Empty-expressing tumors (F, G). (H) The H-scores for Vim in (G) were calculated. The results represent the mean ± SD of four individual tumors per group. The asterisk (*) denotes a statistical significance from Empty and Gata3 samples determined by the T-test. (I, J) 2×10^6^ Empty and Gata3-expressing *p18*^-/-^;*Brca1*^MGKO^ cells were transplanted into the MFPs of NSG mice. When newly generated tumors reached the maximum size allowed by IACUC in 4-10 weeks, or the mice became moribund, lungs were examined for H.E. staining (I) and IHC analysis (insets in I), and quantification of the number of metastatic nodules (J). M, metastatic nodules. Insets in (I) show representative IHC analysis of Vim in lung metastasis. Data in (J) represented as mean ± SD for the numbers of metastatic nodules detected in all lobes of the lungs in each group (n = 4).

Prompted by the finding that Gata3 functions downstream of Brca1 in suppressing EMT in breast cancers, we then examined if ectopic Gata3 in *Brca1*-deficinet tumor cells suppresses their potential for tumor initiation and metastasis. We transduced *p18*^-/-^;*Brca1*^MGKO^ tumor cells with pBabe-Empty and pBabe-Gata3 respectively, and found that ectopic Gata3 restored expression of *Cdh1*, a target of *Gata3*, but inhibited expression of *Vim* and EMT-TFs including *Slug, Snail, and Twist1* (Figure 6A, B), which was stimulated by loss of* Brca1* in mammary tumors (Figure 1A, Figure S1). Morphology analysis revealed that some of the Gata3-expressing *p18*^-/-^;*Brca1*^MGKO^ tumor cells were cuboidal shaped epithelial-like cells whereas all Empty-expressing cells were spindle-shaped, mesenchymal-like cells (Figure 6C). These results suggest that reconstitution of Gata3 in *p18*^-/-^;*Brca1*^MGKO^ tumor cells activates MET. When transplanted, Gata3-expressing *p18*^-/-^;*Brca1*^MGKO^ tumor cells produced significantly smaller tumors with more E-cad but less Vim than Empty-expressing counterparts (Figure 6D, 6F, and Figure S9). Pathological analysis showed that relative to tumors generated by Empty-expressing cells, tumors generated by Gata3-expressing cells were well differentiated with glandular structures, and less aggressive (decreased necrosis, squamous metaplasia, spindle cells, and mitotic indices) (Figure 6G). FACS and IHC analysis revealed that tumors generated by Gata3-expressing *p18*^-/-^;*Brca1*^MGKO^ cells exhibited drastically enhanced CD24-positive and significantly reduced Vim-positive cells relative to those generated by Empty-expressing cells (Figure 6E, G-H, and Figure S9).

To determine if Gata3 affects *Brca1* deficient mammary tumor metastasis, we transplanted Empty- and Gata3-expressing *p18^-/-^; Brca1^MGKO^* tumor cells into MFPs of NSG mice. We discovered that mammary tumors generated by Gata3-expressing cells produced significantly less metastatic nodules in the lung when compared with the mammary tumors initiated by Empty-expressing cells (Figure 6I-J), suggesting that ectopic Gata3 inhibits the metastatic potential of Brca1 deficient mammary tumor cells. In line with these findings, we also observed a drastic reduction of Vim expression in lung metastasis caused by Gata3-expressing cells relative to that in the metastasis done by Empty-expressing cells (Figure 6I, insets). Together, these data suggest that reconstitution of Gata3 activates MET and suppresses *Brca1*-deficient tumor development and metastasis.

## Discussion

In this paper, we found that BRCA1 and GATA3 expressions are positively correlated and that *BRCA1* mutation is associated with the enhanced methylation of the *GATA3* promoter and reduced expression of the *GATA3* gene in human breast cancers. We demonstrated that deletion of *BRCA1* promotes methylation of the *GATA3* promoter, therefore repressing *GATA3* transcription. We discovered that *Gata3* deficiency induces poorly-differentiated mammary tumors with the activation of EMT and promotes the potential for tumor initiation and metastasis. We demonstrated that *Gata3* deficient mammary tumors phenocopy *Brca1* deficient tumors in induction of EMT, and that reconstitution of *Gata3* in *Brca1*-deficient tumor cells activates MET suppressing tumor initiation and metastasis. Our finding demonstrates that GATA3 functions downstream of BRCA1 to suppress EMT in controlling mammary tumor initiation and metastasis.

Functional loss of *BRCA1* is associated with more than half of BLBCs with EMT features 8, 12-14. Though it has been reported that loss of function of *BRCA1* in breast cancers or cell lines enhances the expression of several EMT inducing transcription factors including SLUG 5, TWIST 20, FOXC1/C2 21, and reduces the expression of a few EMT suppressing transcription factors, such as FOXA1 22, 23, FOXO3 24, 25, none of these transcription factors has been identified as downstream mediators of BRCA1 in the regulation of EMT in mammary tumor development and progression. Among the transcription factors upregulated by BRCA1 deficiency, overexpression of these EMT-TF induces EMT, and knockdown of *SLUG, FOXC1,* or* FOXC2* in either *BRCA1* mutant (SUM149 or SUM1315) or* BRCA1* deficient (MDA-MB-231) breast cancer cell lines promotes expression of luminal and epithelial markers *in vitro* 5, 21. For FOXA1 and FOXO3, though overexpression of either of these two transcription factors in cancer cell lines suppresses EMT, loss of either one in mice does not induce EMT in mammary tumor development 23, 25, 59, 60. If a transcription factor primarily functions downstream of Brca1 controlling EMT in breast cancers, genetically loss of or gain of function of this transcription factor in mice with the same genetic background should produce similar EMT phenotype with Brca1 deficient mice, and restoration or removal of the function of the transcription factor should eliminate Brca1 deficient EMT phenotype in mammary tumors, in addition to the regulation of the transcription factor by BRCA1 in vitro. In the present study, we determined and compared the EMT phenotype in mammary tumors developed in mice deficient for* Brca1* or *Gata3* under the same *p18* deficient background. We discovered that *Gata3* deficiency activates EMT in the induction of mammary tumors and promotes tumor initiation as well as the metastatic potential of cancer cells, which phenocopy *Brca1* deficient tumors and tumor cells. We demonstrated that reconstitution of Gata3 in *Brca1*-deficient tumor cells activates MET and eliminates potential for tumor initiation and metastasis. Our finding identifies GATA3 as the first transcription factor that functions downstream of BRCA1 to suppress EMT in breast cancers.

GATA3, a lineage specifier, is critical in controlling the fate of mammary epithelial 10, 11, 61 and lymphoid cells 62. The function of GATA3 in suppressing EMT and metastasis in breast cancers has been well studied in cell line models 19, 26-28. In oncogene transgenic mouse models, loss of *Gata3* marks the loss of tumor differentiation and the onset of tumor dissemination, and deletion of *Gata3* stimulates tumor progression 33, 63. However, due to growth defects induced by long-term loss of *Gata3* and apoptosis caused by acute loss of *Gata3* in differentiated tumor cells 15, 32, 33, it remains elusive if *Gata3* loss regulates EMT in breast cancer development and progression. We and others have previously identified p18 as a downstream target of Gata3 in the control of cell proliferation, and that loss of p18 rescues the proliferative defects induced by Gata3 deficiency 15, 32, 48. *p18;Gata3* double mutant mice provide us a genetic model and a unique opportunity to dissect the role of Gata3 loss in the regulation of tumor cell differentiation in vivo. Taking this advantage in our study, we demonstrated that haploid loss of *Gata3* in *p18* deficient background converts well-differentiated mammary tumors into poorly-differentiated mammary tumors with the activation of EMT and promotes tumor initiating and metastatic potential. Our findings suggest a critical role of Gata3 loss in driving CSC function and metastasis, and support the development of therapeutic drugs enhancing GATA3 function or targeting its downstream pathway to treat metastatic breast cancers.

How GATA3 is regulated is largely unknown. BRCA1 regulates GATA3 expression likely through multiple mechanisms. We and others have demonstrated that BRCA1 binds to GATA3 binding sites on the promoter of a few genes repressing their transcription 20, 21, 64, and that *GATA3* promoter harbors multiple GATA3 binding sites and thus *GATA3* transcription is autoregulatorily activated 65, 66. It is possible that BRCA1 binds to the promoter of *GATA3* to directly activate the transcription of *GATA3*. In addition, since histone methyltransferase EZH2 is overexpressed in *BRCA1*-deficient breast tumor cells 67, and EZH2 binds to *Gata3* promoter repressing its transcription 68, 69, it is also possible that BRCA1 prevents EZH2-mediated H3K27 trimethylation at the *GATA3* locus to maintain *GATA3* transcription. In the present study, we demonstrated that *BRCA1* mutation is associated with *GATA3* promoter hypermethylation and reduced *GATA3* expression in human breast cancer samples. Although deletion of *p18* stimulates expression of DNMT1 in MECs and stromal cells, mice lacking *p18* develop Gata3-positive well-differentiated mammary tumors. Conversely mice deficient for both *Brca1* and* p18* generate Gata3-negative poorly-differentiated tumors in which *Gata3* gene is hypermethylated. These data suggest that when Brca1 is present, methylation of *Gata3* gene in tumor cells is significantly suppressed even if the high level of DNMT1 is available in the tumor and surrounding cells. When Brca1 is depleted, the *Gata3* gene is hypermethylated; likely by DNMT. Our finding that suggests Brca1 protects the *Gata3* promoter from methylation is, at least, one of the important mechanisms by which BRCA1 regulates *GATA3* transcription, though it remains to be investigated that whether BRCA1 directly transactivates* GATA3* and how BRCA1 interacts with DNA methyltransferases (DNMTs) and EZH2 to control transcription of *GATA3*. Our findings also suggest that DNMT inhibitors can be used to induce GATA3 and its mediated differentiation for treatment of *BRCA1*-deficient breast cancers.

## Conclusions

Our finding, for the first time, demonstrates that GATA3 functions downstream of BRCA1 to suppress EMT in controlling mammary tumor initiation and metastasis.

## Supplementary Material

Supplementary figures.

## Figures and Tables

**Table 1:** Spontaneous mammary tumor development in Brca1 mutant mice.

	Genotype ^a^			
Tumor	Wt	*p18*^mt b^	*Brca1*^+/-^	*p18*^mt^;*Brca1^+/-^* ^c^
Mammary Tumor	1/10	23/34 (68%)	1/11	22/31 (71%) ^f^
Metastasis ^d^		2/23 (9%)	0/1	8/22 (36%) ^g^
EMT+ tumor No.^e^		5/23 (22%)	1/1	16/22 (73%) ^h^

^a^ All mice were in Balb/c background and were at 8-22 months of age. ^b^ This group contains eleven *p18^+/-^* and twenty three *p18^-/-^* mice. ^c^ This group contains ten *p18*^+/-^;*Brca1*^+/-^ and twenty one *p18*^-/-^;*Brca1*^+/-^ mice. ^d^ Mammary tumors metastasized mostly to the lung except one to a blood vessel, and one to liver. ^e^ At least two EMT markers (decreased E-Cad, increased Vim, Fn1, SMA or CD29) or two EMT-TFs, which include TWIST, SLUG, SNAIL, FRA1, FOXC1, and FOXC2, were detected in > 2% tumor cells by IHC, as we previously reported (Bai, Cancer Res., 2014). ^f^ No significance from *p18*^mt^;*Brca1*^+/-^ and *p18*^mt^ tumors by a two-tailed Fisher's exact test (p = 0.7948); but p = 0.0008 from *p18*^mt^;*Brca1*^+/-^ and *Brca1^+/-^* tumors by a two-tailed Fisher's exact test. ^g^ A significance from *p18*^mt^;*Brca1*^+/-^ and *p18*^mt^ tumors by a two-tailed Fisher's exact test (p = 0.0351). ^h^ A significance from *p18*^mt^;*Brca1*^+/-^ and *p18*^mt^ tumors by a two-tailed Fisher's exact test (p = 0.0009).

**Table 2:** Spontaneous mammary tumor development in Gata3 mutant mice.

	Genotype ^a^			
Tumor	Wt	*p18*^mt b^	*Gata3*^+/-^	*p18*^mt^;*Gata3*^+/- c^
Mammary Tumor	0/9	8/27 (30%)	0/8	17/34 (50%) ^f^
Metastasis ^d^		0/8		5/17 (29%) ^g^
EMT+ tumor No.^e^		2/8 (25%)		13/17 (77%) ^h^

^a^ All mice were in Balb/c-B6 mixed background and were at 8-22 months of age. ^b^ This group contains eight *p18^+/-^* and nineteen *p18^-/-^* mice. ^c^ This group contains ten *p18*^+/-^;*Gata3*^+/-^ and twenty four *p18*^-/-^;*Gata3*^+/-^ mice. ^d^ Three mammary tumors metastasized to lung, and the other two mammary tumors metastasized to lung and liver. ^e^ At least two EMT markers (decreased E-Cad, increased Vim, Fn1, SMA or CD29) or two EMT-TFs, which include TWIST, SLUG, SNAIL, FRA1, FOXC1, and FOXC2, were detected in > 2% tumor cells by IHC, as we previously reported (Bai, Cancer Res., 2014). ^f^ No significance from *p18*^mt^;*Gata3*^+/-^ and *p18*^mt^ tumors by a two-tailed Fisher's exact test (p = 0.1246). but p = 0.0135 from *p18*^mt^;*Gata3*^+/-^ and *Gata3^+/-^* tumors by a two-tailed Fisher's exact test. ^g^ No significance from *p18*^mt^;*Gata3*^+/-^ and *p18*^mt^ tumors by a two-tailed Fisher's exact test (p = 0.1399). Due to the development of lymphoma and kidney cyst in old *p18*^mt^;*Gata3*^+/-^ mice, we were unable to follow the mammary tumor formation and metastasis in aged mice (mice older than 20 months). ^h^ A significance from *p18*^mt^;*Gata3*^+/-^ and *p18*^mt^ tumors by a two-tailed Fisher's exact test (p = 0.028).

## Abbreviations

BLBC: basal-like breast cancer
Brca1^MGKO^: Brca1^f/f^, MMTV-Cre or Brca1^f/-^, MMTV-Cre
ChIP: chromatin-immunoprecipitation
CSC: cancer stem cell
DAC: 5-aza-2'-deoxycytidine
E-cad: E-cadherin
EMT: epithelial-mesenchymal transition
EMT-TFs: EMT-inducing transcription factors
ER: estrogen receptor
FFPE: formalin-fixed paraffin-embedded
Fn: fibronectin
IHC: immunohistochemistry
INK4: inhibitors of CDK4/6
KD: knockdown
MEC: mammary epithelial cell
MET: mesenchymal-epithelial transition
MFP: mammary fat pad
MS-PCR: methylation specific PCR
p16: p16^INK4A^
p18: p18^INK4C^
TFs: transcription factors
Vim: vimentin
