# Supplementary material for: GATA3 functions downstream of BRCA1 to suppress EMT in breast cancer
Source: Theranostics. 2021 Jul 13;11(17):8218–33. doi: 10.7150/thno.59280 (PMC8344017; doi:10.7150/thno.59280)
Supplement: Supplementary file 1 — Supplementary figures. [file thnov11p8218s1.pdf]

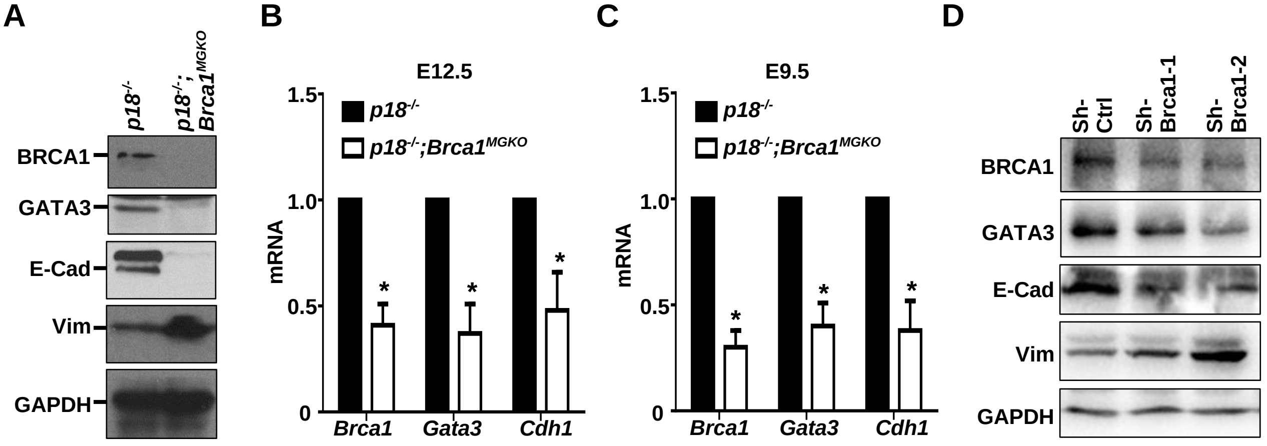

Figure S1. Deletion of *Brca1* reduces the expression of *Gata3* in mouse embryos and cell lines. (A) Primary tumor cells isolated from the mice with the indicated genotype were analyzed by western blot. (B, C) mRNA levels of the indicated genes in *p18*$^{-/-}$ and *p18*$^{-/-}$;*Brca1*^{MGKO} embryos at E12.5 (B) and E9.5 (C) were analyzed by q-RT-PCR. Data represent the mean ± SD from triplicate of each of the two independent embryos. The asterisk (*) denotes a statistical significance from *p18*$^{-/-}$ and *p18*$^{-/-}$;*Brca1*^{MGKO} embryos determined by the T-test. (D) Mouse mammary epithelial cell line, HC11, was infected with pGIPZ-sh-control (sh-Ctrl), as well as pGIPZ-sh-Brca1-1 (sh-Brca1-1) and pGIPZ-sh-Brca1-2 (sh-Brca1-2) targeting different sequences of Brca1. After puromycin selection, cells were analyzed by western blot.

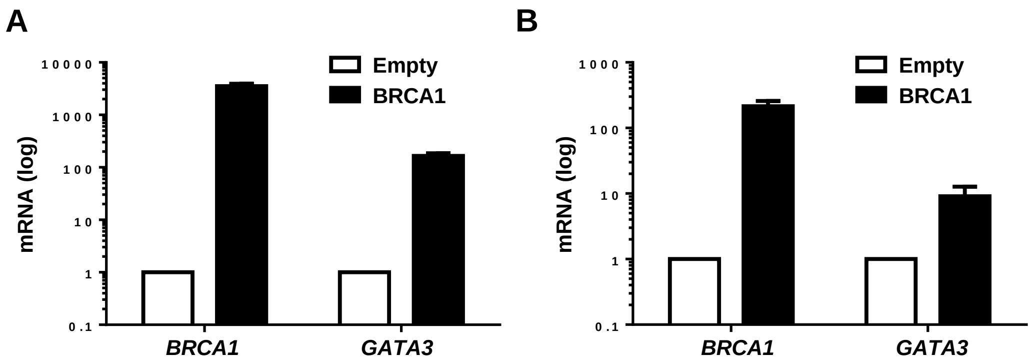

Figure S2. Overexpression of *BRCA1* restore *GATA3* expression in *BRCA1* mutant breast cancer cells. SUM149 (A) and HCC1937 (B) cells were transfected with pBabe-empty (Empty) or pBabe-HA-BRCA1 (BRCA1). Expression of genes indicated were determined by qRT-PCR 48 hours after transfection.

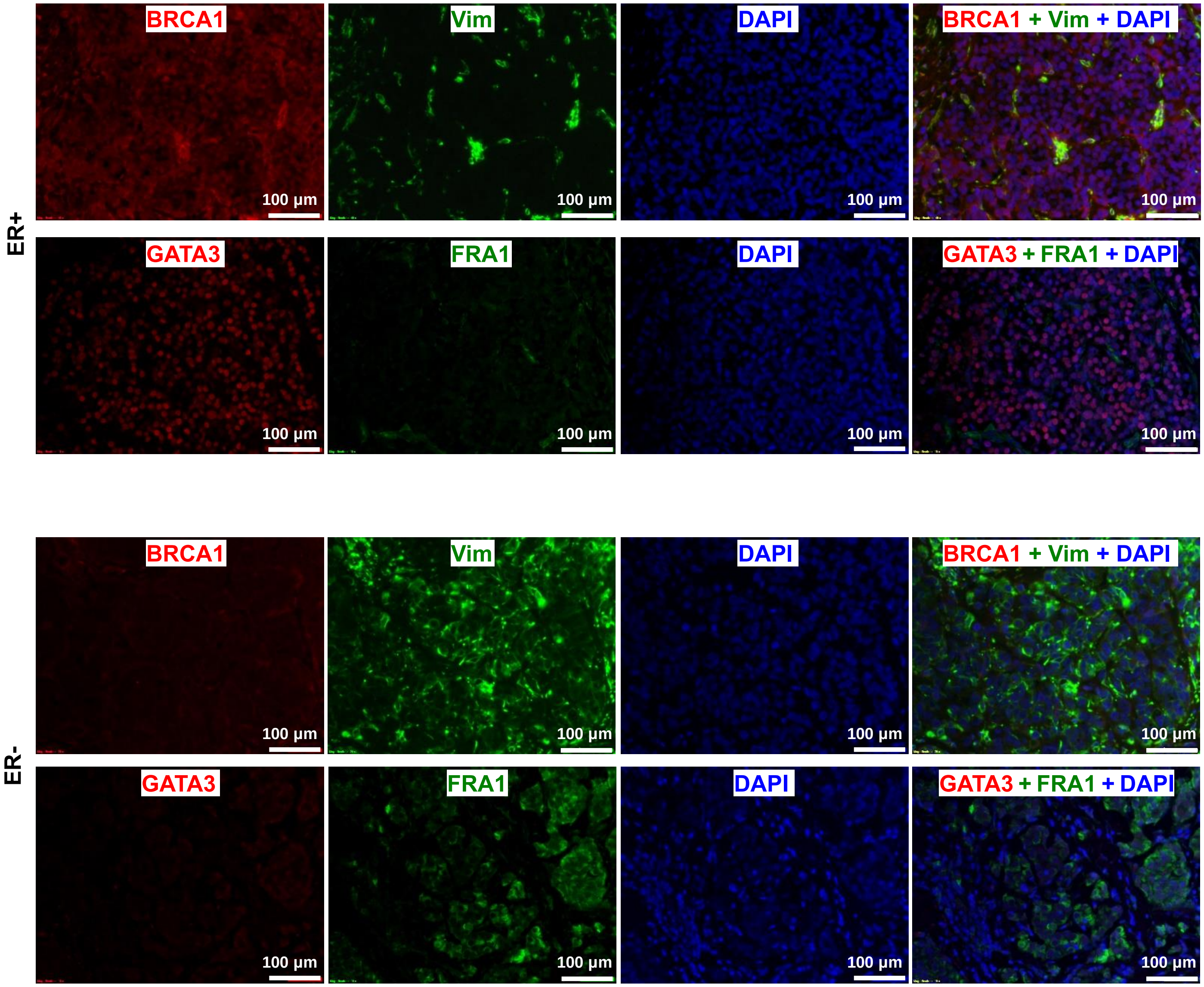

Figure S3. Correlation analysis of GATA3 with BRCA1 in human breast cancers. Representative immunostaining analysis for human breast cancer samples. Case# 8 (ER+) and case#10 (ER-) in Figure 2A were selected for analysis.

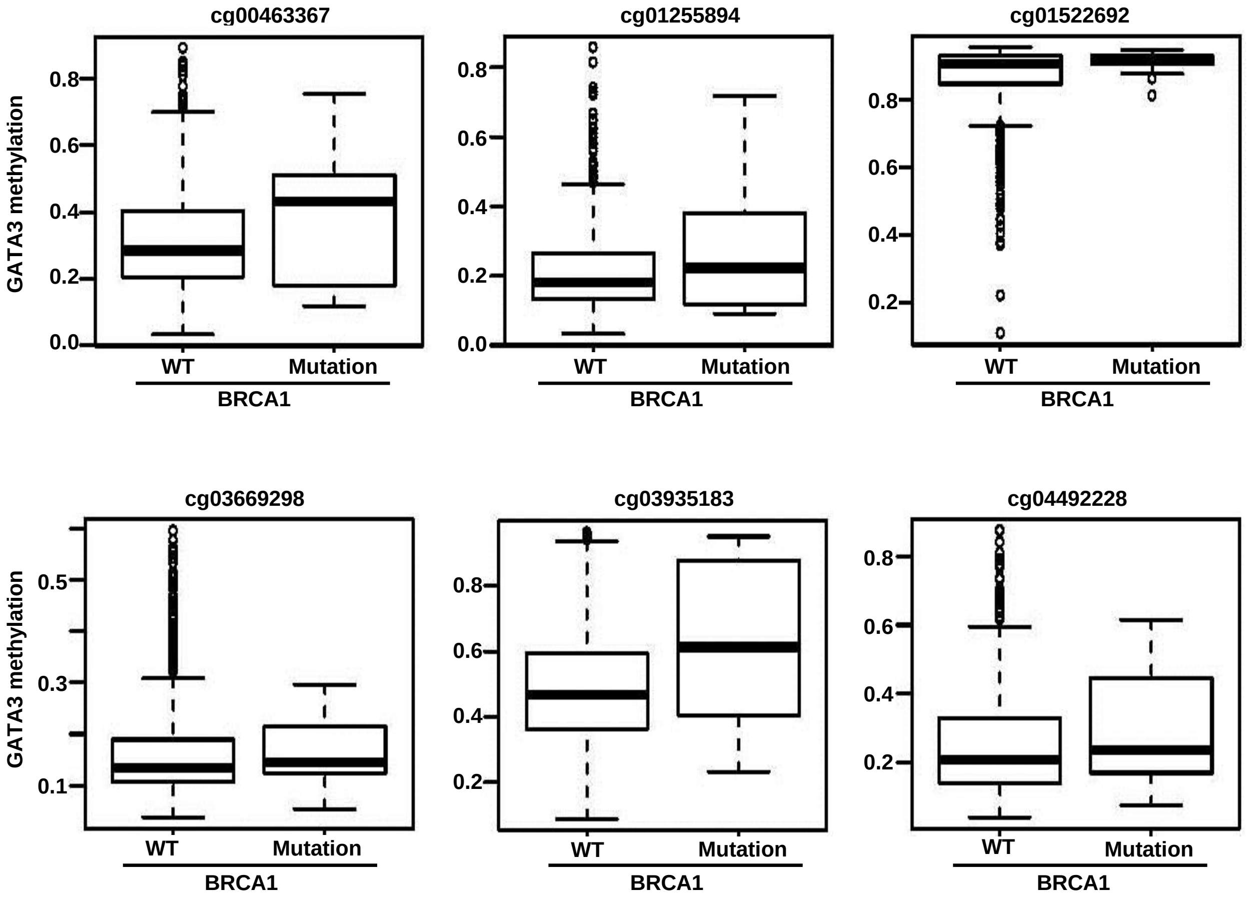

Figure S4. Correlation analysis of GATA3 promoter methylation levels with BRCA1 in human breast cancers. Correlation analysis of GATA3 promoter methylation levels between breast cancers with BRCA1 WT and mutations in the TCGA dataset. Methylation levels in the other 6 CpG sites are shown.

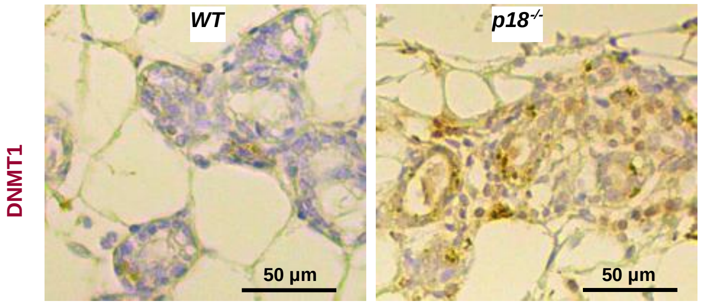

Figure S5. Representative IHC analysis of DNMT1 in virgin mammary tissues.

Note the increase of DNMT1 in p18$^{-/-}$ mammary epithelial cells and stromal cells.

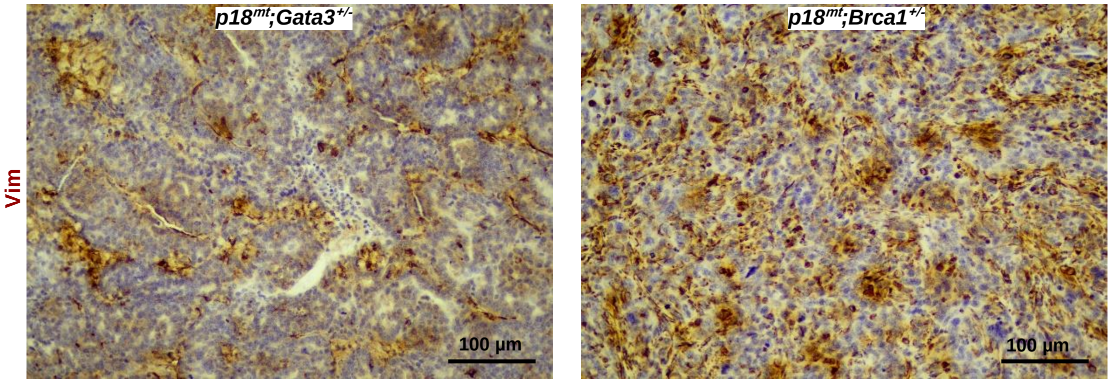

Figure S6. Representative mammary tumors from the indicated genotypes were analyzed by IHC. Note the similarity of the expression pattern of Vim in *p18*^{mt}*;Gata3*$^{+/-}$ and *p18*^{mt}*;Brca1*$^{+/-}$ tumors.

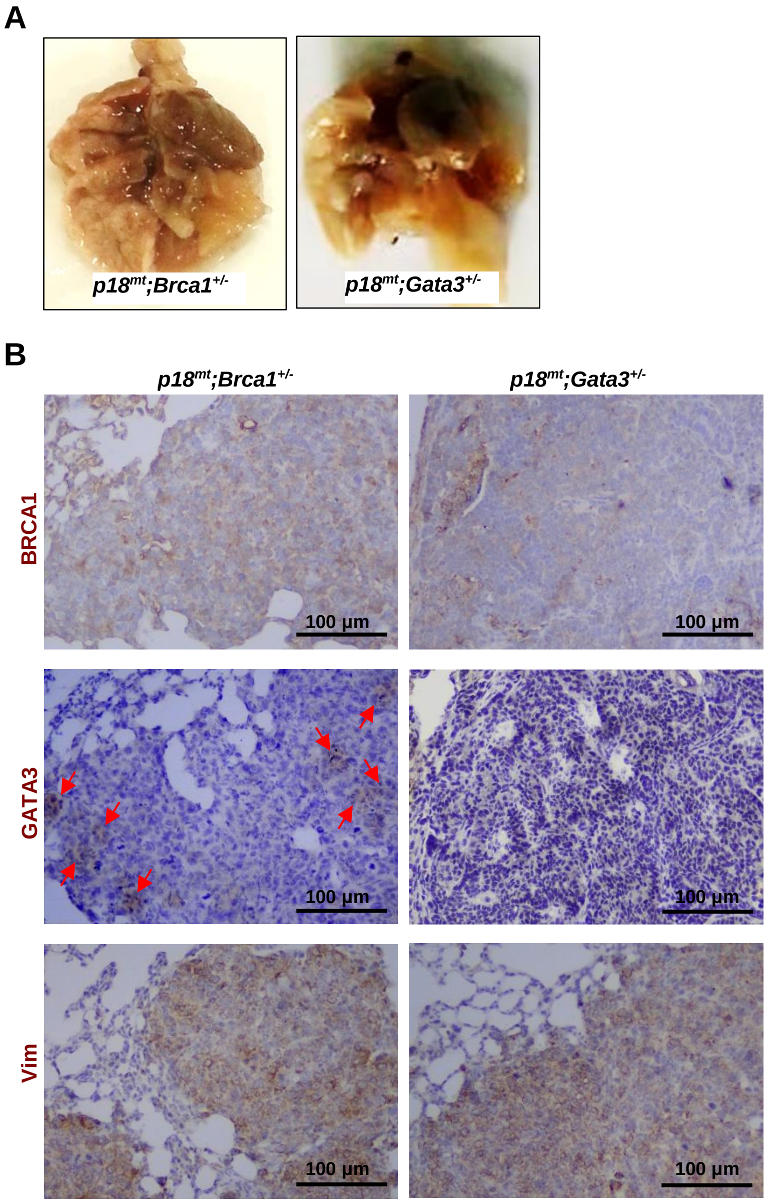

Figure S7. Analysis of spontaneous lung metastasis derived from mouse mammary tumors. (A) Representative gross appearance of the lungs from *p18^{mt};Gata3$^{+/-}$* and *p18^{mt};Brca1$^{+/-}$* mammary tumor bearing mice (B) Representative lung metastases from *p18^{mt};Gata3$^{+/-}$* and *p18^{mt};Brca1$^{+/-}$* mammary tumors were immunostained with antibodies against BRCA1, GATA3, and Vim. Note a few groups of GATA3 weakly-positive tumor cells indicated by red arrows in *p18^{mt};Brca1$^{+/-}$* lung metastases, as well as GATA3 negative tumor cells in *p18^{mt};Gata3$^{+/-}$* lung metastases.

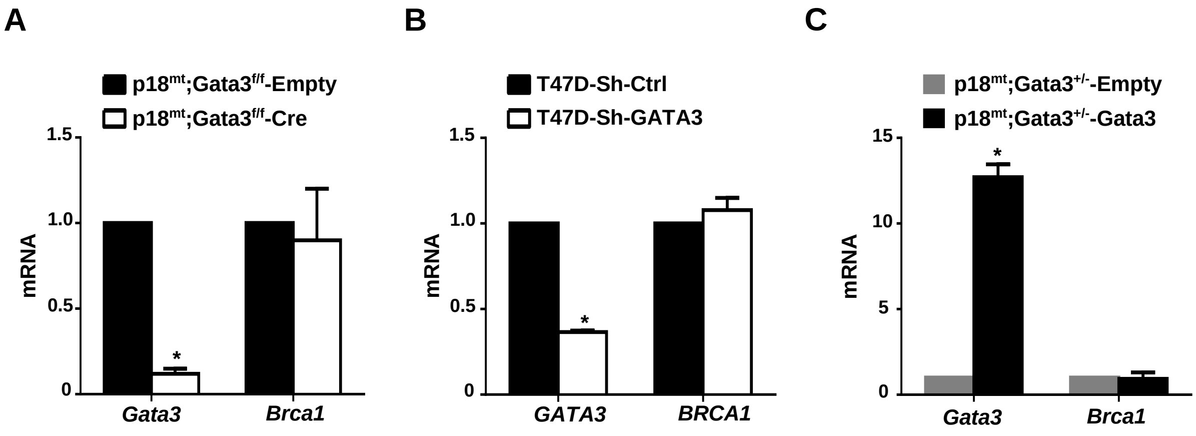

Figure S8. Depletion or overexpression of *Gata3* in mammary cells causes insignificant change of Brca1 expression. (A) *p18^{mt};Gata3$^{f/f}$* mammary epithelial cells were transduced with pMX-Empty (Empty) or pMX-Cre (Cre) and then analyzed by QRT-PCR. Data represent the mean ± SD. from triplicates of two independent *p18^{mt};Gata3$^{f/f}$* cell lines. (B) T47D cells were transduced with pGIPZ-sh-Control (sh-Ctrl) or pGIPZ-sh-GATA3 (sh-GATA3) and then analyzed by QRT-PCR. Data represent the mean ± SD. from triplicates of two independent experiments. (C) *p18^{mt};Gata3$^{+/-}$* mammary tumor cells were transduced with pBabe-Empty (Empty) or pBabe-Gata3 (Gata3) and then analyzed by QRT-PCR. Data represent the mean ± SD. from triplicates of three independent experiments. The asterisk (*) denotes a statistical significance from Cre and Empty, sh-Ctrl and sh-GATA3, or Empty and Gata3 samples determined by the T-test.

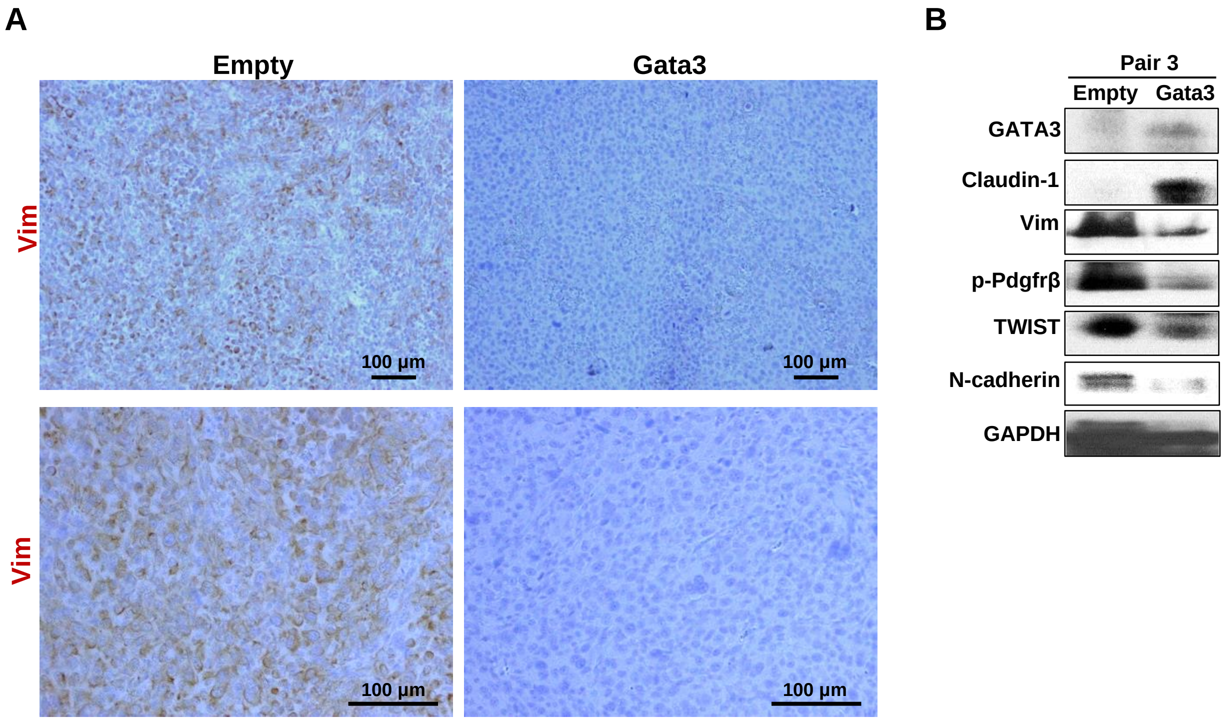

Figure S9. Gata3 activates MET in suppression of Brca1-deficient tumorigenesis. Representative mammary tumors generated by empty- and Gata3-expressing *p18*$^{-/-}$;*Brca1*^{MGKO} tumor cells were analyzed by IHC (A) and western blot (B).
